# Ocriplasmin use in a selected case with preserved visual acuity

**DOI:** 10.1186/s12886-015-0141-9

**Published:** 2015-10-29

**Authors:** Settimio Rossi, Ada Orrico, Paolo Melillo, Francesco Testa, Francesca Simonelli, Michele Della Corte

**Affiliations:** Multidisciplinary Department of Medical, Surgical and Dental Sciences, Eye Clinic, Second University of Naples, Via Sergio Pansini, 5, 80131 Naples, Italy

**Keywords:** Ocriplasmin, Vitreo-macular traction, Multifocal electroretinogram, Optical coherence tomography, Visual acuity

## Abstract

**Background:**

Previous studies described cases of Ocriplasmin injections in patients with vitreo-macular traction and reduced central visual acuity. We describe the first case of a patient with 20/20 visual acuity and vitreo-macular traction treated with Ocriplasmin, and, for the first time in literature, we evaluated the functional changes of the macula in response to pharmacological treatment through multifocal-electroretinogram.

**Case presentation:**

We report the case of a female Caucasian patient aged 67 years with vitreo-macular traction in the right eye, treated with Ocriplasmin, at the Eye Clinic of the Second University of Naples. Visual acuity was 20/20 before treatment, associated with metamorphopsia. Two weeks after injection, optical coherence tomography showed the release of vitreo-macular traction and multifocal electroretinogram responses showed a significant increase of retinal density responses in all six rings (*p* < 0.03). Visual acuity remained constant with resolution of symptoms and the appearance of vitreous floaters.

**Conclusion:**

Intravitreal injection of Ocriplasmin resulted to be a safe and effective treatment in the case here reported. Our data show that the anatomical recovery with release of vitreo-macular traction was associated with a full functional recovery. In fact, the electrical retinal density response of the macular area improved two weeks after Ocriplasmin injection. Further studies with broader inclusion criteria for Ocriplasmin treatment (e.g. also with visual acuity higher than 20/25) on a larger study sample are needed to confirm our results.

## Background

The treatment of vitreomacular interface disorders, in particular vitreomacular adhesion (VMA), vitreomacular traction (VMT), and full-thickness macular hole, has traditionally been limited to either surgical or observational management. Recently, Ocriplasmin (Jetrea; Thrombogenics), a recombinant truncated form of human serine protease plasmin with activity against components of the vitreoretinal interface, including fibronectin and laminin, was approved for the treatment of symptomatic VMA [1]. When injected intravitreally, Ocriplasmin induces vitreous liquefaction and separation of vitreoretinal adhesions at the macula and peripapillary retina [2]. In pivotal phase 3 clinical trials (ClinicalTrials.gov numbers, NCT00781859 and NCT00798317, also referred as MIVI-TRUST clinical trials), patients with a reduced visual acuity (i.e., 20/25 or worse in the eye to be injected) were included and only optical coherence tomography and fluorescein angiography were adopted as outcome measurements [1]. Recently, two case series [3, 4] and two case report [5, 6] of patients treated with intravitreal injection of Ocriplasmin were published. All the patients showed a reduced visual acuity (i.e., 20/25 or worse in the treated eye) and the treatment effects were evaluated by best corrected visual acuity measurement and optical coherence tomography and, in some studies, by standard full-field electroretinography.

We describe the case of a 67-year-old woman with VMT in the right eye, visual acuity of 20/20 and metamorphopsia, treated with intravitreal Ocriplasmin. This is the first time that a patient with preserved visual acuity is treated with Ocriplasmin and that morphological and functional changes of the macula are highlighted using both spectral domain optical coherence tomography (SD-OCT) and multifocal electroretinogram (mf-ERG).

## Case presentation

**Fig. 1:**
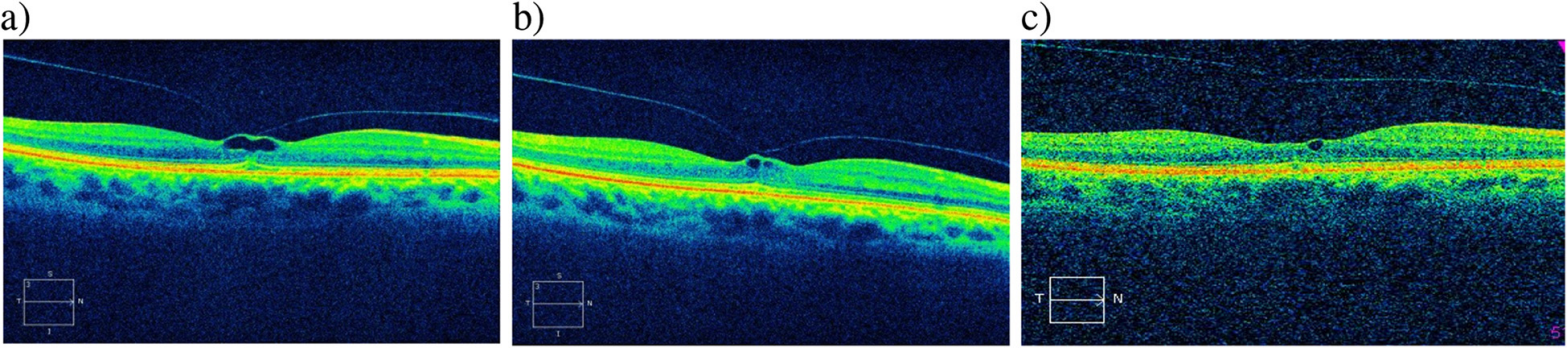
Spectral domain OCT scans before treatment (**a**), one week after treatment (**b**) and two weeks after treatment (**c**). OCT before treatment shows vitreo-macular traction with distortion of the foveal contour and cystoid changes within the retina (**a**); OCT shows changes of retina with persistence of vitreo-macular traction one week after treatment (**b**); OCT shows release of vitreo-macular with a small cystic alteration two weeks after treatment (**c**)

We present a clinical case of a 67-year-old-woman with VMT in the right eye. Two years previously, she underwent vitrectomy in the left eye for macular hole at the Eye Clinic of the Second University of Naples. On examination, the patient referred metamorphopsia in the right eye. A complete ophthalmic examination was performed, including best-corrected visual acuity (BCVA) by Snellen visual chart, slit lamp biomicroscopy, fundus examination, SD-OCT and mf-ERG before treatment, and at one and two weeks after intravitreal Ocriplasmin injection in the right eye. BCVA was 20/20 in the right eye. Fundus examination and SD-OCT revealed VMT with distortion of the foveal contour and cystoid changes within the retina in the right eye. The inner segment-outer segment (ellipsoid) photoreceptor layer was altered for morphology and reflectivity in the central portion corresponding to the VMT in the right eye (Fig. 1a).

**Fig. 2:**
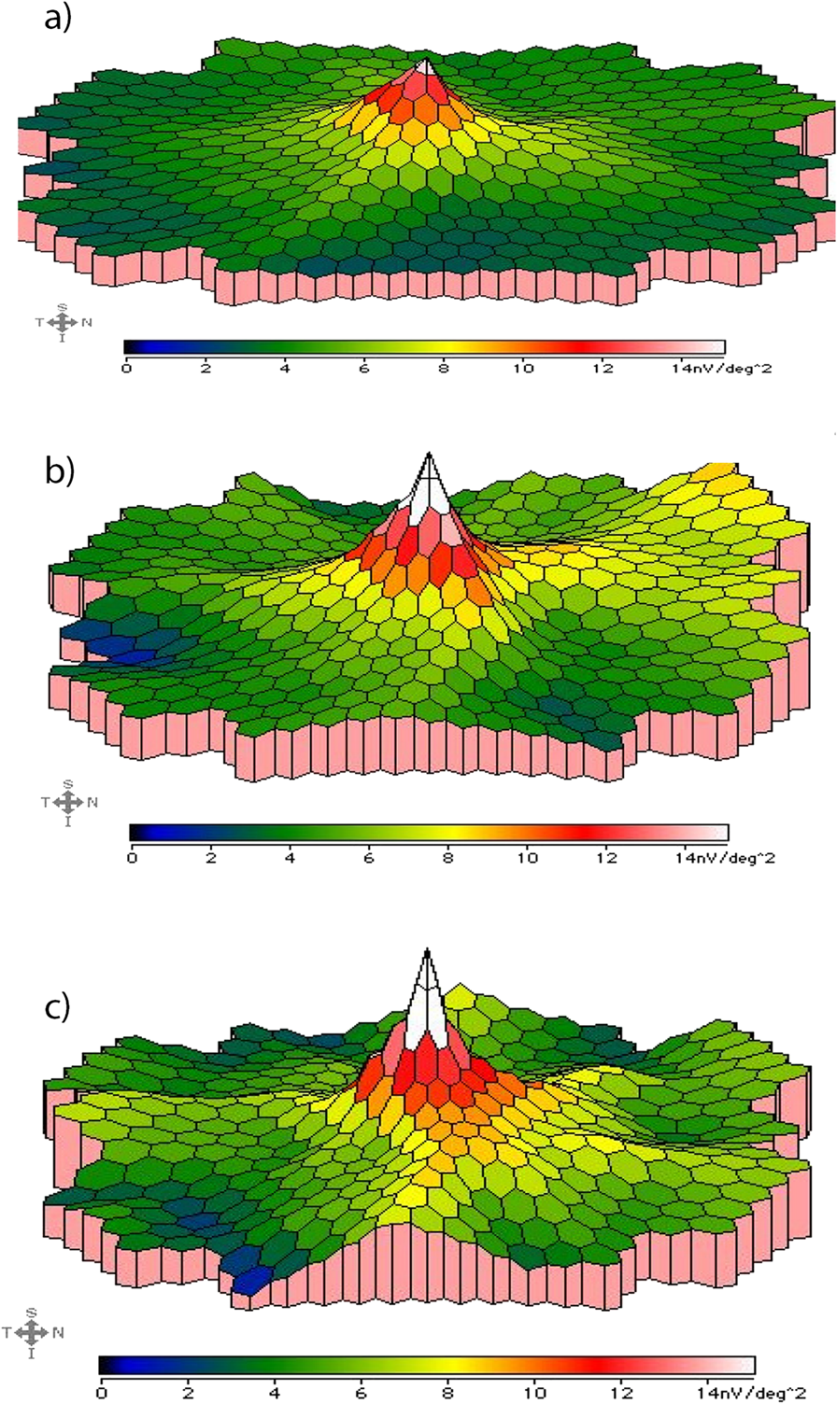
Multifocal electroretinogram responses before (**a**), one week after treatment (**b**) and two weeks after treatment (**c**). Multifocal electroretinogram shows a significant increase of the foveal peak density response of the treated eye one week and two weeks after pharmacological treatment

One week after intravitreal injection of Ocriplasmin (0.125 mg/0.1 mL) in the right eye, BCVA was stable to 20/20 in the injected eye. SD-OCT demonstrated changes of retina with persistence of VMT and alteration of the ellipsoid layer (Fig. 1b) while mf-ERG revealed an increased foveal peak density response in the treated eye (Fig. 2a, b).

**Fig. 3:**
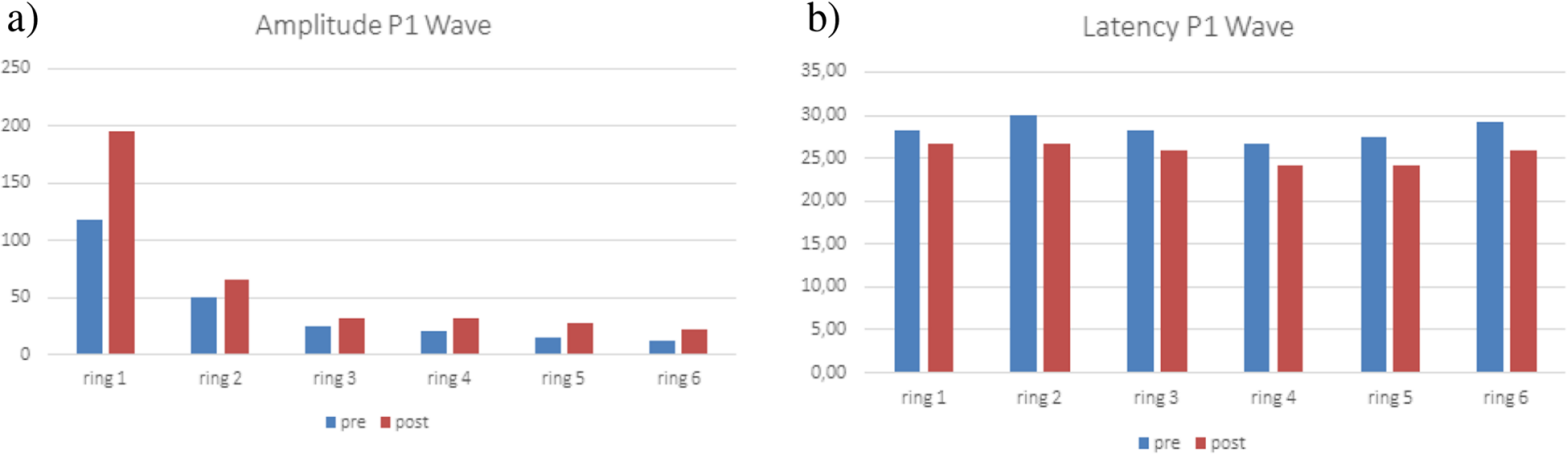
Amplitude (**a**) and latency (**b**) of P1 wave amplitudes in the six rings of multifocal electroretinogram before and after treatment. Multifocal electroretinogram shows a significant increase of P1 wave amplitude and a significant reduction of latency in all six rings two weeks after intravitreal Ocriplasmin

Two weeks after treatment, we observed VMT release, stable visual acuity and a preserved foveal profile showing a small cystic alteration and a continuous ellipsoid layer in right eye (Fig. 1c); mf-ERG revealed a further increase in foveal peak density response in the treated eye (Fig. 2). Visual acuity remained stable in the treated eye. The patient had no metamorphopsia while vitreous floaters were observed in the right eye. Furthermore, we observed an increase of P1 wave amplitude (Fig. 3a) in all six rings of mf-ERG responses compared to the pre-treatment examination in the treated eye. We also noticed a reduction between pre- and post-treatment timepoints, shifting towards normal ranges, in the treated eye (Fig. 3b). The longitudinal analysis (linear regression model fitted to log-transformed data, with ring as covariate) showed a significant increase of P1 wave at an estimated exponential rate of 4.2 % per week (*p* < 0.001) and a significant reduction of the latency at an estimated exponential rate of 0.4 % per week (*p* = 0.009).

## Discussion

Our study analyzes the first case of Ocriplasmin treatment in a patient with VMT and 20/20 visual acuity. Our patient satisfied all inclusion and exclusion criteria of MIVI-TRUST clinical trials [1], with the only exception of preserved visual acuity in the treated eye (i.e. BCVA of 20/20). The patient complained of metamorphopsia before Ocriplasmin injection. After pharmacological treatment her visual acuity remained stable with the appearance of vitreous floaters.

Recent case reports [5, 6] have described a transient decrease of visual acuity in treated eyes. One case report [6] described a 71-year-old woman with symptomatic VMT who experienced persistent darkening of vision for four months after receiving intravitreal Ocriplasmin. The symptoms of darkened vision corresponded to the disruption of the inner segment-outer segment (ellipsoid) photoreceptor layer and reduced a- and b-wave full-field ERG amplitudes. However, this worsening in visual function persisted in spite of the immediate release of VMT. The other study [5] reported a case of persistent visual loss associated with acute severe panretinal dysfunction after Ocriplasmin injection for a small macular hole with VMA. This was associated with Goldmann visual field constriction, anisocoria, attenuated retinal vessels, disruption of outer retinal signals on SD-OCT, and severely reduced full-field ERG responses.

Our patient maintains a visual acuity of 20/20 but refers vitreous floaters after treatment. The release of VMT is documented through OCT. The morphological analysis showed an alteration of the ellipsoid layer only in the portion below the VMT with resolution following its release.

Above all, our work shows that the anatomical result coincides with the functional improvement recorded by mf-ERG. The density response analysis demonstrated a significant increase in the foveal peak of the treated eye after injection compared to baseline testing. Moreover, mf-ERG responses showed a significant increase of P1 wave amplitude and a reduction of latency in all six rings compared to baseline values. mf-ERG reflects the electrophysiological response of cones, but also those of the inner retinal layers [7]. VMT release induced by Ocriplasmin injection probably leads to the restoration of the nerve fiber layer, the ganglion cell layer and the foveal Muller cells. In the case report by Tibbetts et al. [6], mf-ERG was performed but the results obtained were not discussed. In particular we highlight that the release of VMT leads not only to the recovery of macular morphology but also to the restoration of macular function.

## Conclusion

Intravitreal injection of Ocriplasmin was a safe and effective treatment in our case, characterized by a preserved visual acuity. Our data show that the anatomical recovery with release of VMT is associated with a full functional recovery, without visual acuity loss. In fact, the electrical retinal density response of the macular area improved two weeks after Ocriplasmin injection. Appropriate attention to details is important in patient selection as such selection of suitable candidates could determine good outcomes following treatment with Ocriplasmin. Since the present study reports only one case, further studies on an adequate sample are needed in order to approve broader selection criteria including subjects with higher visual acuity.

## Consent

Written informed consent was obtained from the patient for publication of this Case report and any accompanying images. A copy of the written consent is available for review by the Editor of this journal.
